# Severe Dietary Zinc Deficiency Does Not Significantly Alter Energy Balance in Adult Mice

**DOI:** 10.1155/jnme/6911386

**Published:** 2025-08-25

**Authors:** Caitlin C. Murdoch, Andy Weiss, Kyle T. Enriquez, Kacie A. Traina, Sydney L. Drury, Nathan C. Winn, Louise L. Lantier, Eric P. Skaar

**Affiliations:** ^1^Department of Pathology, Microbiology, and Immunology, Vanderbilt Institute for Infection, Immunology and Inflammation, Vanderbilt University Medical Center, Nashville, Tennessee, USA; ^2^Department of Medicine, Vanderbilt University Medical Center, Nashville, Tennessee, USA; ^3^Department of Molecular Physiology and Biophysics, Vanderbilt University, Nashville, Tennessee, USA; ^4^Vanderbilt Mouse Metabolic Phenotyping Center, Nashville, Tennessee, USA

**Keywords:** energy balance, glucose tolerance, metabolic cages, mice, zinc deficiency

## Abstract

Zinc (Zn) is an essential dietary nutrient metal that functions as a cofactor for numerous enzymes involved in diverse cellular processes, including energy metabolism. In humans, Zn deficiency afflicts an estimated one-third of the global population and is a prominent risk factor for numerous diseases, including the development of obesity and diabetes. It is known that severe Zn deficiency leads to impaired growth and development in animals, suggesting that this dietary micronutrient is required for the maintenance of organismal energy balance. However, the impact of Zn restriction on energy balance, specifically energy intake versus expenditure, remains incompletely described in existing murine models of Zn deficiency. Here, we characterized the impact of a prolonged Zn-restricted diet on animal growth, energy balance, and glucose metabolism using metabolic cage analysis and oral glucose tolerance tests in adult mice. While we demonstrated dietary Zn-dependent reductions in body weight with concomitant decreases in energy expenditure and energy intake, we found no significant alterations in energy balance. Furthermore, we observed modest sex-dependent impacts on glucose tolerance. Collectively, these data highlight that prolonged, severe Zn restriction in adult mice does not elicit significant changes in metabolic parameters such as overall energy balance and glucose clearance. These findings indicate that other factors lead to the changes in body weight and composition in Zn-deficient mice.

## 1. Introduction

Zinc (Zn) is an essential dietary micronutrient that functions in numerous organismal and cellular processes, thereby exerting a significant impact on human health [1, 2]. Despite the vital requirement of Zn, the World Health Organization estimates that the global prevalence of Zn deficiency is as high as 31%, rendering inadequate Zn intake as a major public health threat [3, 4]. The leading cause of Zn deficiency is malnutrition and insufficient dietary intake; however, inherited genetic mutations as well as chronic illnesses can also give rise to impaired Zn uptake and utilization [5, 6]. Disruptions in Zn homeostasis have been linked to a spectrum of human disorders, including growth retardation, obesity, cancers, and neurodegeneration [2].

The importance of Zn is in large part attributable to its role as a structural and catalytic cofactor for approximately 10% of proteins in humans, many of which regulate diverse cellular processes including proliferation, transcription, and metabolism [7]. Numerous metabolic enzymes involved in the synthesis of carbohydrates, proteins, and lipids require Zn as a cofactor. Furthermore, Zn regulates the synthesis, storage, and release of insulin [8, 9]. It is therefore unsurprising that Zn imbalance has been implicated in metabolic syndromes including obesity, atherosclerosis, insulin resistance, and Type 2 diabetes [9, 10]. In humans, serum Zn levels are inversely correlated with body mass index (BMI) and directly correlated with metabolic biomarkers such as glycemic status and plasma levels of cholesterol and insulin [11, 12]. It is also thought that dysregulated Zn levels contribute to the progression of metabolic disease because Zn reduces oxidative stress and inflammatory cytokine production [13]. As such, Zn supplementation has been found to mitigate symptoms of metabolic disease as well as malnutrition in humans, representing a promising dietary intervention to circumvent pathology derived from Zn deficiency [11, 14, 15]. Together, these properties highlight Zn as a critical trace nutrient essential for maintaining and supporting metabolic health.

Although it is established that Zn deficiency leads to growth impairments, reductions in lean body mass, as well as obesity, the factors leading to these observed changes remain incompletely understood [16]. The maintenance of a healthy body weight in response to environmental changes, such as inadequate Zn intake, is regulated in large part through energy metabolism, or the conversion of food to energy [17]. Therefore, an organism's energy balance—determined by the combination of energy intake and expenditure—plays a crucial role in mediating changes in body weight and composition [18]. Numerous reports suggest that dietary Zn intake impacts energy balance in humans. Studies illustrate that Zn deficiency is associated with fatigue and Zn supplementation can lead to increases in physical activity [19–22]. Moreover, Zn deficiency has been linked to decreases in appetite [23].

To investigate the impact of severe, prolonged Zn deficiency on energy balance, we developed a dietary model of Zn deficiency wherein adult mice are administered a low Zn (< 1 ppb) diet for 6 weeks. In this study, we found that diet-induced Zn deficiency altered body composition while restricting animal growth, food and fluid intake, and activity. Despite these changes, this model of Zn deficiency resulted in no profound alterations in overall energy balance, indicating that decreases in body weight likely result from differences in tissue deposition and the reduction in lean body mass.

Finally, due to the vital role of Zn in supporting the function of numerous metabolic enzymes and insulin-dependent cell signaling, we further sought to understand how long-term Zn limitation impacts response to glucose. In adult mice, dietary Zn limitation exhibited minimal impact on the glucose tolerance of female mice, while a slightly elevated insulin sensitivity was observed in male mice as compared to control-fed counterparts. Together, these data indicate that dietary Zn is required for growth with severe deficiency contributing to altered body composition and attenuated body weight. However, the concomitant decreases in both energy intake and expenditure lead to an overall maintenance of energy balance.

## 2. Methods

### 2.1. Mouse Strains, Husbandry, and Diet

Experiments were performed using six-week-old male and female C57BL/6J (Jackson Laboratories) mice. Animals were maintained at the Vanderbilt University Medical Center Animal Facilities and housed in groups of five. The exact age and sex of animals used for each individual experiment are denoted in the corresponding figure legends. For routine colony maintenance, mice were fed a standard chow diet (LabDiets; Rodent Chow Diet 5L0D PicoLab). For manipulation of organismal Zn levels, mice were fed a defined Zn-free or control diet (Dyets Inc., AIN-93M) and a purified Zn-free (< 1 ppb) rodent diet with or without Zn supplementation at 29 parts per million. Defined diets were administered at 6 weeks of age and maintained for 6 weeks. For experimental endpoints, animals were humanely euthanized. Mouse studies were approved by the Institutional Animal Care and Use Committees of Vanderbilt University Medical Center (protocol number M1900043-01) in accordance with the Public Health Service Policy on the Human Care and Use of Laboratory Animals under the Unites States of America National Institutes of Health (NIH) Office of Laboratory Animal Welfare (OLAW).

### 2.2. Indirect Calorimetry and Ambulatory Activity

Indirect calorimetry and ambulatory activity studies were performed as previously described [24]. Briefly, a standard 12 h light/dark cycle was maintained throughout the calorimetry studies. Female mice, after acclimation to individual housing for 7 days, were placed in metabolic cages located in the Mouse Metabolic Phenotyping Center at Vanderbilt University (RRID: SCIR_021939) in a temperature- and humidity-controlled dedicated housing room. All animals had *ad libitum* access to control or low zinc diet and water throughout the study. Respiratory gases, locomotor activity, and feeding were determined using the Promethion Metabolic Analyzer (Sable Systems, North Las Vegas, NV). Energy expenditure was calculated from V̇O2 and V̇CO2 using the Weir equation: Kcal/hr = 60 × (0.003941 × VO_2_ + 0.001106 × VCO_2_) [25]. The respiratory exchange ratio (RER) was calculated as the ratio of CO_2_ production (VCO_2_) over O_2_ consumption (VO_2_). Ambulatory activity was determined simultaneously every second with the collection of the calorimetry data. Ambulatory activity and position were detected with XYZ beam arrays (BXYZ-R, Sable Systems, Las Vegas, NV) with a beam spacing of 1.0 cm interpolated to a centroid resolution of 0.25 cm. Consecutive adjacent infrared beam breaks were counted and converted to distance, with a minimum movement threshold set at 1 cm. Data acquisition and instrument control were coordinated by MetaScreen, and the obtained raw data were processed using MacroInterpreter (Sable Systems, Las Vegas, NV) using an analysis script detailing all aspects of data transformation. The script is available on request from Sable Systems.

### 2.3. CalR

Metabolic cage data were visualized using the web-based tool, CalR (https://www.CalRapp.org/). Data were uploaded in the two-group format. For statistical analysis, body weight was used as the covariate.

### 2.4. Body Composition

Body composition was determined by NMR (Bruker Minispec).

### 2.5. Determination of Metal Levels by Inductively Coupled Plasma Mass Spectrometry (ICP-MS)

Murine tissues were homogenized in PBS supplemented with 1% IGEPAL using a Bullet Blender (Next Advance) at 4°C. Mouse tissue homogenates were acid digested in 400 μL of Optima grade nitric acid (Thermo Fisher Scientific) and 100 μL of Ultratrace hydrogen peroxide (Sigma) at 65°C for 48 h. After digestion, the acid content was diluted to below 10% with 3.5 mL of UltraPure water (Invitrogen). For the collection of serum samples, whole blood was extracted via heart needle puncture. Samples were placed immediately on ice, after which they were spun down to extract sera from other whole blood fractions. Matched volume of sera was immediately stored in 15-mL VWR metal-free tubes, diluted to 1 mL with metal-free Ultrapure water (Invitrogen). Elemental quantification was performed using an Agilent 7700 ICP-MS (Agilent) attached to a Teledyne CETAC Technologies ASX-560 autosampler (Teledyne CETAC Technologies). The following settings were fixed for the analysis: cell entrance = −40 V, cell exit = −60 V, plate bias = −60 V, OctP bias = −18 V, and collision cell helium flow = 4.5 mL/min. Optimal voltages for Extract 2, Omega Bias, Omega Lens, OctP RF, and Deflect were determined empirically before each sample set was analyzed. Element calibration curves were generated using ARISTAR ICP Standard Mix (VWR, BDH82026-108). Samples were introduced by peristaltic pump with 0.5-mm-internal-diameter tubing through a MicroMist borosilicate glass nebulizer (Agilent). Samples were initially taken at 0.5 rps for 30 s followed by 30 s at 0.1 rps to stabilize the signal. Samples were analyzed in spectrum mode at 0.1 rps collecting three points across each peak and performing three replicates of 100 sweeps for each element analyzed. Sampling probe and tubing were rinsed for 20 s at 0.5 rps with 2% nitric acid between every sample. Data were acquired and analyzed using the Agilent Mass Hunter Workstation Software Version A.01.02.

### 2.6. Oral Glucose Tolerance Tests (OGTTs)

After a 5-h fast in a home cage with paper-based bedding, tails of mice were nicked and baseline samples taken for glucose (2 μL) and insulin (20 μL). Samples for measurement of glucose concentrations were taken at *t* = 10, 20, 30, 45, 60, 90, and 120 min after a glucose gavage (2 g/kg whole body). Plasma samples for insulin measurements were also taken at *t* = 0, 10, and 30 min after the glucose bolus. At the conclusion of the study, mice were returned to their home cages.

### 2.7. Statistical Analysis

**Figure 1:**
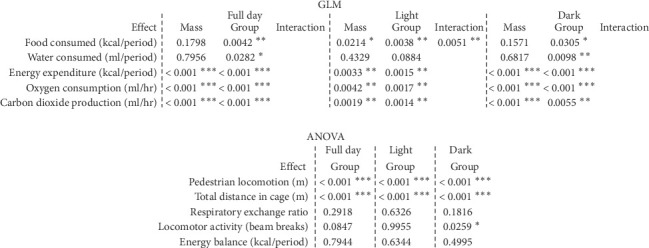
Weight-adjusted statistical analysis of diet-dependent metabolic measures via CalR. For metabolic cage data from weeks four to five, CalR was used to determine the appropriate statistical model. For mass-dependent variables, the body mass variable was specified and included as a covariate. CalR performs the appropriate statistical model, starting with a general linearized model (GLM). Data were either analyzed with an ANOVA (no covariate), ANCOVA (ANOVA + covariate), or ANOVA with interaction. Measurements not associated with mass (e.g., pedestrian locomotion and respiratory exchange ratio) were analyzed by a one-way ANOVA. *N* = 8/diet. ^∗^*p* < 0.05, ^∗∗^*p* < 0.01, ^∗∗∗^*p* < 0.001.

Raw data were collected in Microsoft Excel and imported into GraphPad Prism or CalR for visualization and statistical analysis [26]. For each set of comparisons, either a Student's *t*-test, one-way ANOVA with Tukey's correction for multiple comparisons, or two-way ANOVA with Tukey's or Sidak's multiple comparison post-test was performed based upon the sample size, expected distribution of sample values, and the statistical question being asked. The individual test used per figure subpanel is denoted in the figure captions. For metabolic cage data, CalR was used to determine the appropriate statistical model [26] (Figure 1, Supporting Figure 4). For mass-dependent variables, the body mass variable was specified and included as a covariate. CalR internally determined and performed the appropriate statistical model, starting with a general linearized model (GLM). Data were either analyzed with an ANOVA (no covariate), ANCOVA (ANOVA + covariate), or ANOVA with interaction (Figure 1, Supporting Figure 4). For measurements not associated with mass (e.g., pedestrian locomotion and RER), the difference between groups was analyzed by a one-way ANOVA. The alpha value associated with significance was *p* < 0.05 and denoted as follows: ^∗^*p* < 0.05, ^∗∗^*p* < 0.01, ^∗∗∗^*p* < 0.001, ^∗∗∗∗^*p* < 0.0001. Sample sizes are indicated in the figure legends. Exact statistical tests used, group sizes, and dispersion and precision of measurements are defined for each panel within the figure legends.

## 3. Results

### 3.1. Zn-Deficient Mice Exhibit Growth Defects and Changes in Body Composition

**Figure 2:**
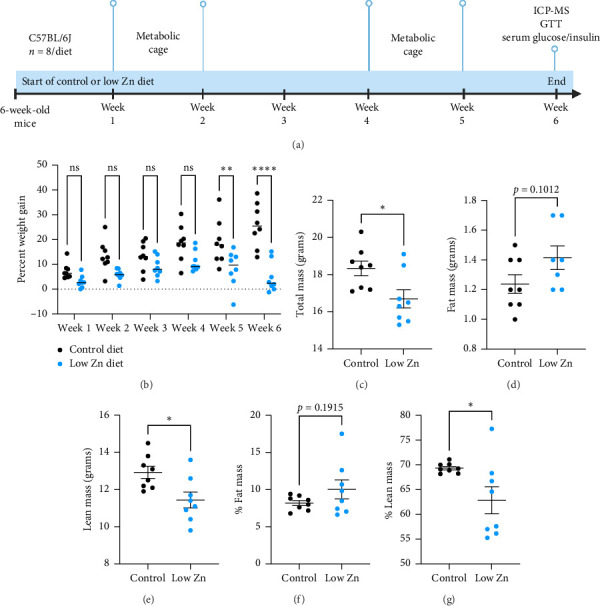
Body composition and metabolic caging analysis in low Zn diet-fed mice. (a) Experimental schematic of dietary Zn restriction in six-week-old mice. *N* = 8/diet. (b) Percent weight gain as compared to week zero of mice on a control or low Zn diet for 6 weeks. (c) Total mass of mice at the end of metabolic cage evaluation at week 5. (d–g) Quantification of body composition ((d and f)—fat mass; (e and g)—lean mass) of mice fed a control or low Zn diet at week 5. All data are represented as mean ± SEM. Statistical significance in panel (b) measured by two-way ANOVA with Sidak's multiple comparison test. Statistical significance in panels (c–g) determined by Student's *t*-test. ^∗^*p* < 0.05, ^∗∗^*p* < 0.01, ^∗∗∗^*p* < 0.001, ^∗∗∗∗^*p* < 0.0001.

To interrogate the metabolic effects of severe Zn deficiency in adult mice, six-week-old mice were placed on either a control or a low Zn diet, wherein Zn levels were restricted to nearly undetectable amounts (> 1 ppb) to most potently limit Zn availability, for a period of 6 weeks (Figure 2(a)). Control diet, by comparison, was prepared from a low Zn diet supplemented with 29 ppm of Zn. Previous studies in our laboratory and others have demonstrated that Zn deficiency in animals leads to defects in growth [27–29] and changes in body composition [16]. Indeed, mice on a low Zn diet gained significantly less weight as compared to mice on a control diet and began losing weight after 5 weeks of Zn restriction (Figure 2(b)). To identify the factors that underly the Zn-dependent reduction in weight (Figures 2(b) and 2(c)), body composition was analyzed using nuclear magnetic resonance after 4 weeks of a low Zn diet. While there was no significant change in fat mass (Figure 2(d)), mice on a low Zn diet had reduced lean mass as compared to mice on a control diet (Figure 2(e)). Considering the reduced body weight of Zn-deficient mice, these data revealed that mice on a low Zn diet have significant reductions in percent lean mass and a modest increase in percent fat mass (Figures 2(f) and 2(g)). To further evaluate if changes in lean body mass were dependent on changes in organ size, dissected tissues were weighed from control and Zn-deficient mice. This revealed no significant reductions in tissue weights in mice fed a low Zn diet, indicating that the observed decrease in lean mass may be due to reduced muscle or bone mass (Supporting Figure 1). Together, these data demonstrate that a prolonged Zn-deficient diet reduces growth in adult mice, with a specific reduction in lean mass.

### 3.2. Systemic Metal Levels Are Altered in States of Zn Deficiency

**Figure 3:**
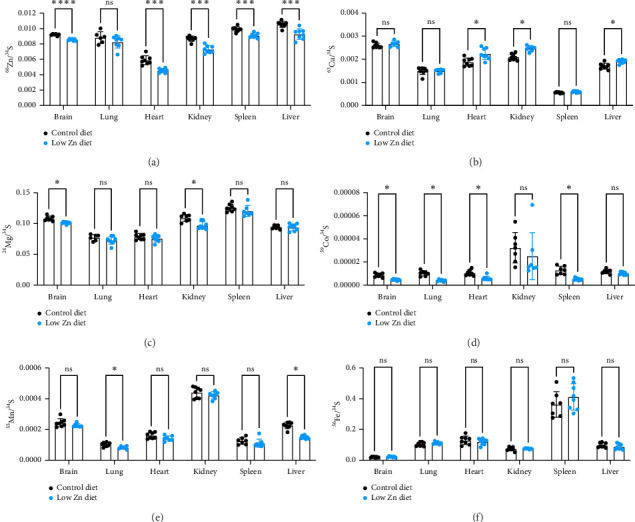
Systemic metal levels are altered in mice fed a Zn-deficient diet. (a–f) ICP-MS quantification of metal levels from dissected tissues of mice fed a control or low Zn diet for 6 weeks. *N* = 8/diet. All data are represented as mean ± SEM. Statistical significance determined by Student's *t*-tests. ^∗^*p* < 0.05, ^∗∗^*p* < 0.01, ^∗∗∗^*p* < 0.001, ^∗∗∗∗^*p* < 0.0001.

To test the impact of this murine model of dietary Zn restriction on systemic metal abundance, organ metal levels were quantified from mice fed a control or low Zn diet using ICP-MS. As expected, levels of Zn were significantly reduced in most tissues, including the brain, heart, kidney, liver, and spleen (Figure 3(a)). Evaluation of other metals indicated tissue-specific increases in copper (Cu) as well as decreases in cobalt (Co), magnesium (Mg), and manganese (Mn) in organs from mice fed a Zn-restricted diet (Figures 3(b), 3(c), 3(d), 3(e), 3(f)). These data demonstrate that dietary restriction of Zn not only reduced Zn levels in mice but more broadly influenced the abundance of other metals in a tissue-specific manner, raising the possibility that decreased Zn abundance alters the absorption, excretion, and/or storage of other metals. To further test if Zn deficiency alters the intestinal absorption of other metals, metal levels from feces and serum were quantified. No significant changes in metal levels were observed in serum in mice fed a low Zn diet compared to control (Supporting Figure 2). In feces, a significant reduction of Zn was measured, while the abundance of other metals was largely unchanged. Together, these data suggest that this model of Zn limitation does not significantly alter the absorption of other metals from diet (Supporting Figure 2).

### 3.3. Metabolic Factors Dictating Energy Balance Are Dysregulated in Zn-Deficient Mice

To understand the metabolic and behavioral differences that underly the growth impairments in Zn-deficient mice, mice fed a control or low Zn diet were placed in metabolic cages (Sable Systems) following 1 and 4 weeks of diet to evaluate organismal energy homeostasis (Figure 2(a)). Metabolic cage analysis provides extensive and detailed data on energy expenditure (CO_2_/O_2_ detection and physical activity monitoring) as well as energy input (food and water consumption). Measurements from these experiments can be used to calculate energy balance, a metabolic parameter that is maintained through the coordinated regulation of energy intake (i.e., food consumption) and energy expenditure (i.e., physical activity). Analysis of these data indicated that there were no significant alterations in energy expenditure in Zn-deficient mice after 1 week of diet (Supporting Figure 3), yet perturbations in energy intake and output emerged after prolonged Zn starvation (Figure 1, Supporting Figure 4).

**Figure 4:**
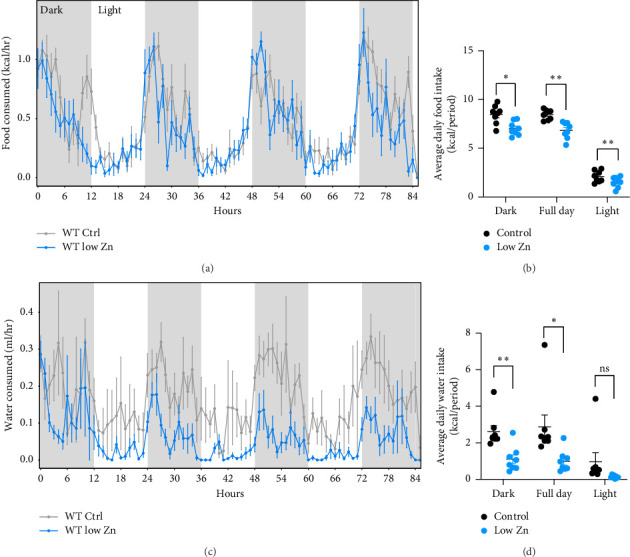
Mice fed a Zn-deficient diet exhibit a modest reduction in food and fluid intake. (a–d) Quantification of food (a and b) and water (c and d) consumption over a five-day period (panels (a and c): light phase—white shading, dark phase—gray shading). *N* = 8/diet. All data are represented as mean ± SEM. Statistical significance in panels (b and d) was determined by the generalized linear model in CalR. ^∗^*p* < 0.05, ^∗∗^*p* < 0.01, ^∗∗∗^*p* < 0.001.

**Figure 5:**
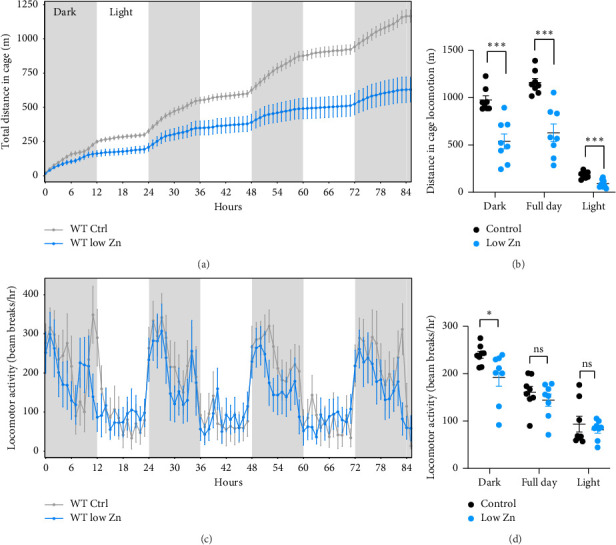
Physical activity is reduced in mice fed a low Zn diet. (a and b) Total distance traveled and (c and d) beam breaks over a five-day period (Panels (a and c): light phase—white shading, dark phase—gray shading). *N* = 8/diet. All data are represented as mean ± SEM. Statistical significance in panels (b and d) was determined by the ANOVA model in CalR. ^∗^*p* < 0.05, ^∗∗^*p* < 0.01, ^∗∗∗^*p* < 0.001.

**Figure 6:**
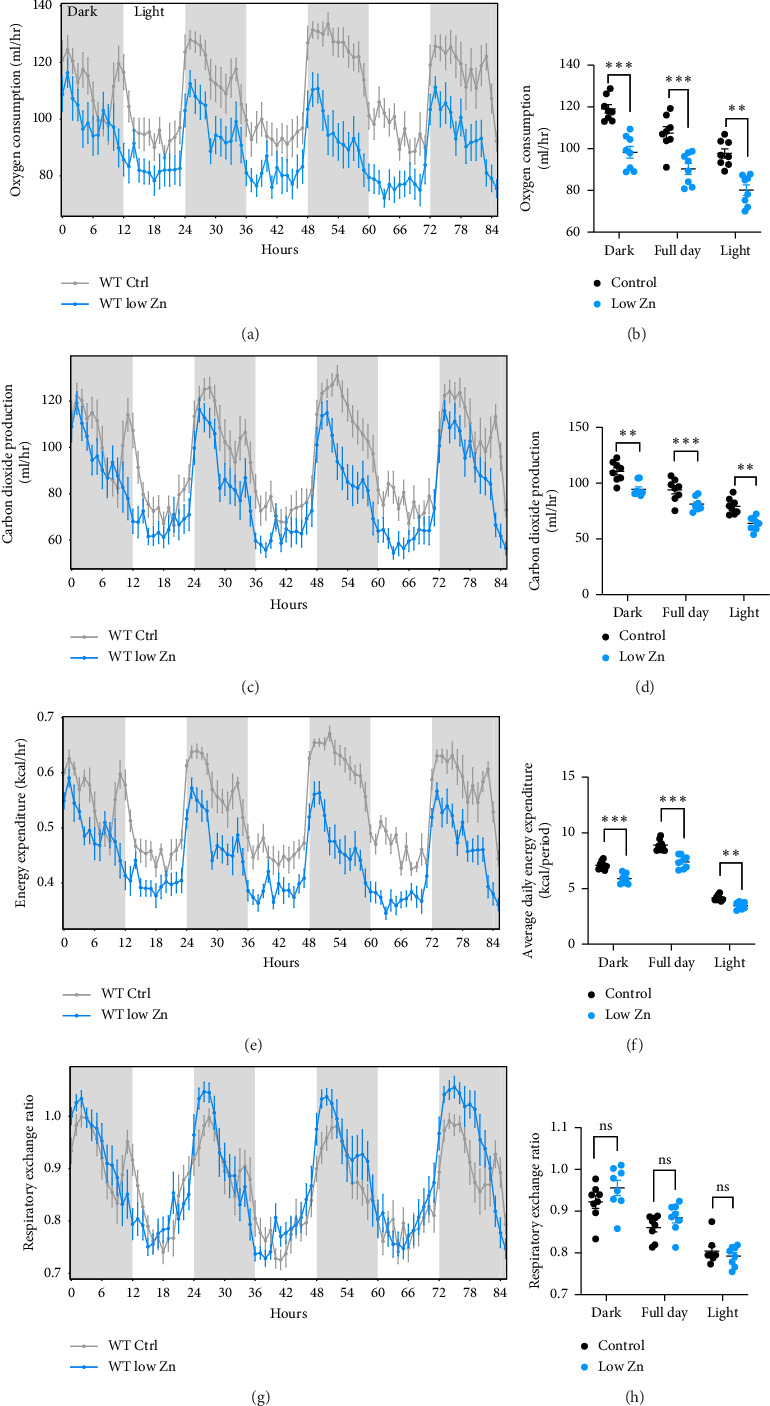
Zn-deficient mice have reduced energy expenditure. (a and b) Oxygen consumption and (c and d) carbon dioxide production were measured to evaluate energy expenditure (e and f) and respiratory exchange ratio (g and h) in Zn-deficient mice (Panels (a, c, e, and g): light phase—white shading, dark phase—gray shading). *N* = 8/diet. All data are represented as mean ± SEM. Statistical significance in panels (b, d, and f) was determined by generalized linear model as described in Figure 1 and Supporting Figure 4 with total mass as the covariate and panel H by one-way ANOVA in CalR. ^∗^*p* < 0.05, ^∗∗^*p* < 0.01, ^∗∗∗^*p* < 0.001.

Analysis of food consumption revealed that Zn-deficient mice exhibited a modest reduction in the food consumed particularly during the light phase (Figures 4(a) and 4(b)), with a concomitant decrease in water intake (Figures 4(c) and 4(d)). To evaluate the effect of Zn deficiency on energy output, physical activity was monitored, O_2_ consumption and CO_2_ production were measured, and energy expenditure was calculated. Mice fed a Zn deplete diet exhibited significantly reduced activity as compared to controls as evaluated by total distance traveled in the cage (Figure 5). Additionally, CO_2_ production and O_2_ consumption were quantified to indirectly measure heat production and energy expenditure. Zn-deficient mice exhibited both attenuated O_2_ consumption and CO_2_ production (Figures 6(a), 6(b), 6(c), 6(d)) compared to mice fed a control diet, indicating that Zn deficiency leads to reduced energy expenditure (Figures 6(e) and 6(f)). To more fully understand the metabolic profile of mice on a low Zn diet, the RER was calculated. RER is an index of whole-body relative substrate utilization, with an RER of 1 indicating maximal reliance on carbohydrate oxidation and an RER of 0.7 indicating maximal reliance on fatty acid oxidation. In healthy standard chow-fed mice, RER will oscillate between these two values in a distinct circadian pattern. No significant differences were observed, suggesting that Zn deficiency does not strongly change the overall proportion of carbohydrate and fatty acid being oxidized (Figures 6(g) and 6(h)).

Measurements of energy expenditure and energy intake were then used to quantify energy balance. Although Zn-deficient mice have similar energy balance as compared to animals on a control diet (Supporting Figure 5), each of the metabolic parameters underlying this balance is impacted by Zn deficiency. Notably, mice on a low Zn diet expend less energy while also taking less energy in via food and fluid intake, potentially underlying the failure of these animals to gain weight and build lean mass as compared to mice on a control diet. Cumulatively, these data reveal that Zn deficiency results in changes to the metabolic profile of mice, including a reduction in energy output, yet energy balance is maintained due to a concomitant reduction in food intake.

### 3.4. Glucose Metabolism is Modestly Dysregulated in Adult Male Zn-Deficient Mice

**Figure 7:**
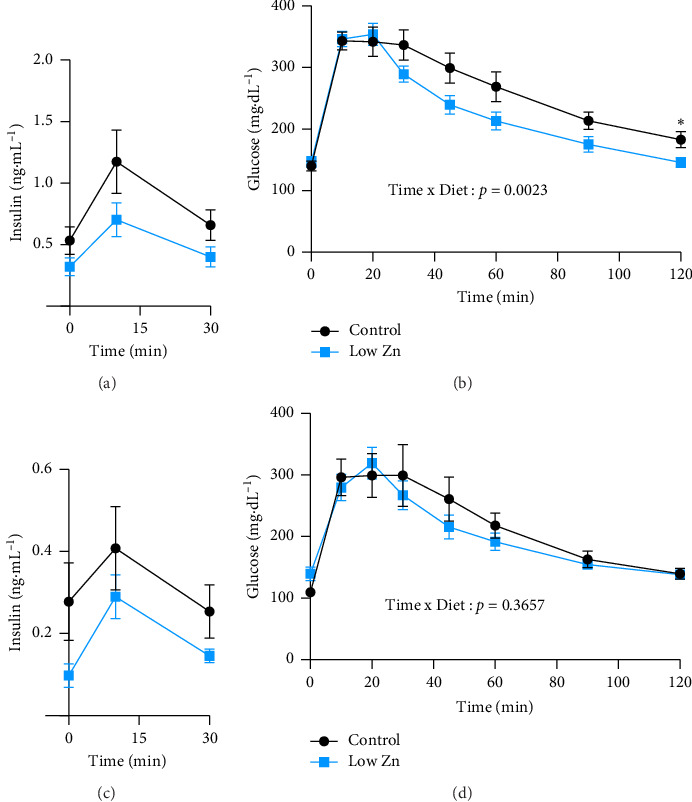
Sex-dependent alterations in glucose clearance in Zn-deficient mice. (a and b) Quantification of insulin (a) and glucose (b) levels from male mice following oral administration of glucose. *N* = 10/diet. (c and d) Quantification of insulin (c) and glucose (d) levels from female mice following oral administration of glucose. *N* = 5/diet. All data are represented as mean ± SEM. Statistical significance in panels (a–d) was measured by RM two-way ANOVA with Geisser–Greenhouse correction and Tukey's multiple comparison test. ^∗^*p* < 0.05, ^∗∗^*p* < 0.01, ^∗∗∗^*p* < 0.001, ^∗∗∗∗^*p* < 0.0001.

Given that Zn deficiency is associated with impaired glucose homeostasis and insulin resistance [2, 9] and Zn-deficient mice have reduced body weight (Figure 2(b)), glucose homeostasis was evaluated in control and Zn-deficient mice using OGTTs. Although serum insulin levels were modestly reduced in Zn-deficient mice as compared to controls (Figures 7(a) and 7(c)), no significant changes in glucose clearance were detected in female mice (Figure 7(d)). Interestingly, glucose clearance was increased in male Zn-deficient mice compared to control diet-fed counterparts despite reduced levels of insulin (Figures 7(a) and 7(b)). These results suggest that this model of Zn restriction does not lead to drastic impairments in glucose tolerance, yet reveals sex-dependent alterations in insulin sensitivity in male mice.

## 4. Discussion

Zn is an essential micronutrient that is required for the maintenance of cellular and organismal homeostasis. Since Zn cannot be synthesized, it must be obtained through dietary sources. Importantly, Zn is not stored at high levels within cells and tissues, and therefore, constant intake of this nutrient metal is required to support animal health. Zn is vital for numerous functions including proper growth and tissue development, reproduction, wound healing, and mounting proper immune responses [1, 5, 6]. Despite the broad physiological importance of Zn, it is estimated that between 17% and 31% of the world's population is at risk of Zn deficiency, largely due to poor dietary intake [1, 3, 4]. Notably, estimates of the prevalence of Zn deficiency can be complex and highly variable due to the scarcity of biomarkers and adequate measurements of dietary Zn intake [30]. Distinct populations of individuals have an elevated risk of Zn deficiency, particularly those living in developing countries who face food insecurity and malnutrition. However, other factors including age, pregnancy, inherited genetic mutations, and chronic illnesses can impact Zn status and can change the recommended levels of daily Zn intake [2, 6].

Zn functions as a structural and catalytic cofactor for over 300 different enzymes, thereby enabling their catalytic activity while also participating in signaling as free ions [2, 3, 8, 13]. Due to this property, Zn is involved in many cellular activities such as transcription, DNA repair, signaling, cellular proliferation, and the synthesis of new proteins. Zn deficiency has been identified as a risk factor for numerous human pathologies, ranging from cancers, neurodegeneration, and metabolic syndromes such as obesity and diabetes [2]. Thus, inadequate Zn intake represents a major global health threat.

Zn is required for the growth of bones and tissues, thereby promoting childhood and adolescent growth as well as the conservation of body mass in adults [16]. Interestingly, studies suggest long-lasting impacts of Zn restriction on metabolic health, as children who have experienced stunting due to Zn deficiency are at higher risk for developing obesity later in life [31]. The vast majority of Zn in the body is stored in skeletal muscle and bone [32]. Shifts in body composition, including lean and fat mass, are associated with changes in Zn levels. Specifically, lean body mass is rich in Zn and has a high Zn requirement for synthesis. As such, lean mass is often reduced in conditions of low Zn both in people and animal models including this study [14, 16, 33, 34].

Given that regulation of body weight is driven by energy metabolism and energy balance, it is likely that Zn levels contribute to organismal energy homeostasis [17]. To this end, Zn status is associated with physical activity (energy expenditure) and feeding behaviors (energy intake) in rodent models and humans [20, 23, 35–38]. However, the impact of prolonged severe Zn restriction on whole-animal energy homeostasis is not fully understood, highlighting the need for the development of in vivo animal models of Zn limitation.

To interrogate the impact of Zn on organismal health, we developed a dietary model of Zn deficiency in adult mice wherein six-week-old C57BL6/J mice were maintained on a low Zn or a control diet for 6 weeks [28, 39]. This level and duration of Zn restriction result in several host phenotypes, including defects in whole animal growth (Figure 2(b)) and increased susceptibility to infection by bacterial pathogens, suggesting immunologic defects [39, 40]. Given that Zn-deficient animals consistently fail to gain weight (Figure 2(b)), we sought to understand the broad metabolic impact of Zn deficiency in adult mice. In this study, we evaluated organismal energy metabolism using metabolic cage analysis coupled with glucose tolerance tests.

Zn-deficient mice failed to gain weight over 6 weeks and exhibited a significant reduction in lean body mass with no changes in fat mass (Figures 2(d) and 2(e)). These findings are consistent with observations in humans that Zn status is correlated with lean body mass [16]. Indeed, in a rat model of Zn deficiency, animals exhibited a decreased lean body mass accompanied with an increase in body fat mass [16]. Reductions in lean mass of Zn-deficient mice may be associated with the decrease in physical activity recorded during the metabolic cage studies (Figure 5). Additionally, attenuated protein synthesis, a hallmark of Zn deficiency, may also contribute to observed alterations in body composition [16, 41]. It is also likely that other factors, including appetite-regulating hormones such as leptin, may underlie this growth impairment during Zn limitation [23, 38, 42]. To this end, we observed a modest reduction in food consumption in Zn-deficient animals, suggesting potential Zn-dependent impacts on satiety (Figure 4). Reduced food intake has been frequently reported in Zn-deficient humans and rats [23, 35].

It has been shown that manipulation of specific dietary metal levels can interfere with the absorption, excretion, transport, and storage of other metals [43]. In humans, Zn deficiency has been associated with alterations in serum levels of other trace metals and this trend has also been observed in rodent models [43]. In line with these observations, this model of dietary Zn deficiency resulted in alterations of other trace metals, notably Co, Mg, Mn, and Cu in a tissue-specific manner (Figure 3). These data suggest that specific tissue stores of metals respond to variations in dietary Zn at different rates. Interestingly, we observed no significant changes in fecal metal levels, apart from Zn, between mice on a control or low Zn diet both at 1 week and 5 weeks following the onset of diet (Supporting Figure 2). This suggests that absorption of other metals was likely not significantly altered in Zn-deficient mice, raising the possibility that there were Zn-dependent impacts on tissue absorption, excretion, or storage of other metals. Of note, the Cu/Zn ratio was elevated in Zn-deficient animals with increased Cu levels in tissues including the heart, kidney, and liver (Figure 3(b)). It is well established that this ratio is associated with oxidative and nutritional stress and can modulate host immune defense, expression of growth factors, and stress [44], thereby implicating Zn deficiency in the dysregulation of systemic organismal responses.

Mice on a low Zn diet have decreased energy expenditure, with significant reductions in physical activity (Figure 5). These findings are consistent with other studies demonstrating fatigue in Zn-deficient humans and activity in rats [19, 34, 45]. Interestingly, the RER in adult Zn-deficient mice indicated a potential alteration in carbon sources (Figures 6(g) and 6(h)). Based on bomb calorimetry, it is known that an RER of 1.0 is 100% carbohydrate oxidation; an RER of 0.7 is pure lipid oxidation. However, in Zn-deficient mice, the RER exceeds 1.0 during the early dark phase (Figure 6(h)). This implies that new carbon, potentially fat, was being generated raising the possibility that these animals were compensating for low energy expenditure by increasing nutrient stores *de novo* [46–48]. In fact, increased lipogenesis has been reported in Zn-deficient rats [49–51]. Moreover, the fat mass of Zn-deficient mice was not decreased despite lower lean mass, and a percentage of total body weight (% fat) was modestly increased. During the light cycle (fasting), nutrient stores are mobilized to fuel tissue metabolism and the troughs in RER were not different between control and Zn-deficient mice, suggesting that nutrient stores were similarly mobilized between groups.

Despite differences in energy expenditure and body composition, glucose clearance in this model of Zn deficiency was unchanged in female mice but slightly increased in Zn-deficient male mice versus controls in a setting of reduced insulin tone (Figure 7). This finding suggests that Zn restriction leads to improved insulin sensitivity in male mice. In part, this mirror published reports on Zn-deficient rats where there was no change in glucose response [52, 53]. Notably, Zn-deficient adult mice fed a high-fat diet also exhibited no increases in adiposity, systemic inflammation, or metabolic function as measured by glucose tolerance [54]. However, in a genetic model of Zn deficiency where animals harbor a mutation in the Zn transporter, *Znt8*, glucose intolerance due to elevated insulin degradation was observed, suggesting that genetic changes leading to dysregulated Zn metabolism do not mimic a dietary deficiency of the metal [9, 55]. In our model of Zn deficiency, the observed reduction in insulin levels is unlikely to be due to defects in insulin deflation/clearance, given that glucose tolerance is improved (Figure 7). It is more likely that the beta-cells have adapted to the glucose-tolerant environment and are simply producing the appropriate levels of insulin to maintain glucose homeostasis.

Overall, this study demonstrates no significant changes in energy balance, with broad reductions in both energy intake and expenditure in Zn-starved mice. Additionally, these findings highlight the importance of broadly evaluating organismal phenotypes in different models of Zn deficiency. It is likely that timepoint of induction, duration, and magnitude of Zn insufficiency result in distinct phenotypic outcomes. Thus, defining the organismal phenotypes of Zn deficiency should be interrogated within each model to fully understand the manifestation of symptoms as it relates to Zn deficiency in humans. While we report metabolic defects, including attenuated energy expenditure resulting from dietary Zn restriction, we do not recapitulate all phenotypes observed in Zn-deficient humans or other rodent Zn deficiency models. It is possible that more moderate models of Zn deficiency for longer timescales may more accurately reflect the physiology of chronic Zn deficiency in humans. Moreover, the combination of additional dietary or genetic models of metabolic stress, such as a high-fat diet or leptin mutations, may be necessary to evaluate metabolic dysfunction in the context of Zn deficiency.

## 5. Conclusions

A prolonged model of severe Zn deficiency in adult mice resulted in a hypometabolic state with modest sex-dependent alterations in glucose tolerance. Our findings suggest that failure of Zn-deficient mice to gain body weight is associated with changes in body composition, reduced energy intake, and decreased energy expenditure.

## Data Availability

All raw data are included within the manuscript and available from the corresponding author upon request.

## Nomenclature

Zn: Zinc
ICP-MS: Inductively coupled plasma mass spectrometry
OGTT: Oral glucose tolerance test

## Supporting Information

Supporting Figure 1. Organ weights from low Zn diet-fed mice. Organs were harvested and weighed following 6 weeks of control or low Zn diet. *N* = 10/diet. All data are represented as mean ± SEM. Data in panels A–F analyzed by Student's *t*-test. ^∗^*p* < 0.05, ^∗∗^*p* < 0.01, ^∗∗∗^*p* < 0.001, ^∗∗∗∗^*p* < 0.0001.

Supporting Figure 2. Fecal and serum metal levels of mice fed a control or low Zn diet. Metal levels quantified from control or Zn-deficient mice from feces (A-H) after one and 5 weeks of dietary intervention and serum (I) after 6 weeks of low Zn diet. Feces: *N* = 8/diet, serum: *N* = 4/diet. All data are represented as mean ± SEM. Data in panels A–H analyzed by two-way ANOVA with Tukey's multiple comparisons test. Data in panel I analyzed by Student's *t*-tests. ^∗^*p* < 0.05, ^∗∗^*p* < 0.01, ^∗∗∗^*p* < 0.001, ^∗∗∗∗^*p* < 0.0001.

Supporting Figure 3. Metabolic cage analysis following 1 week of low Zn diet. (A) Energy expenditure, (B) locomotor activity, and (C) food consumption were measured to evaluate (D) energy balance in Zn-deficient mice following 1 week of diet (panels A–D: light phase—white shading, dark phase—gray shading). *N* = 8/diet. All data are represented as mean ± SEM. (E) Weight-adjusted statistical analysis of diet-dependent metabolic measures via CalR.

Supporting Figure 4. GLM-based regression plots. CalR analysis of group by mass interactions for (A) oxygen consumption, (B) energy expenditure, (C) carbon dioxide production, and (D) food consumption for mice in metabolic cages from weeks four to five of diet. *N* = 8/diet. Slopes of control or low Zn-fed groups were used to determine appropriate statistical model. For variables that exhibit group-dependent mass interactions as demonstrated by slopes that are dissimilar between groups, ANOVA with interaction was performed. Otherwise, data were analyzed using GLM/ANCOVA.

Supporting Figure 5. Energy balance of Zn-deficient mice. (A-D) Energy balance was quantified from measurements of energy expenditure and energy intake (panels A and C: light phase—white shading, dark phase—gray shading). *N* = 8/diet. All data are represented as mean ± SEM. Statistical significance in panels B and D was determined by ANOVA model in CalR.
