# Supplementary material for: Severe Dietary Zinc Deficiency Does Not Significantly Alter Energy Balance in Adult Mice
Source: J Nutr Metab. 2025 Aug 25;2025:6911386. doi: 10.1155/jnme/6911386 (PMC12401606; doi:10.1155/jnme/6911386)
Supplement: Supporting Information — Additional supporting information can be found online in the Supporting Information section. [file 6911386.f1.docx]

**Supplemental Materials**

**
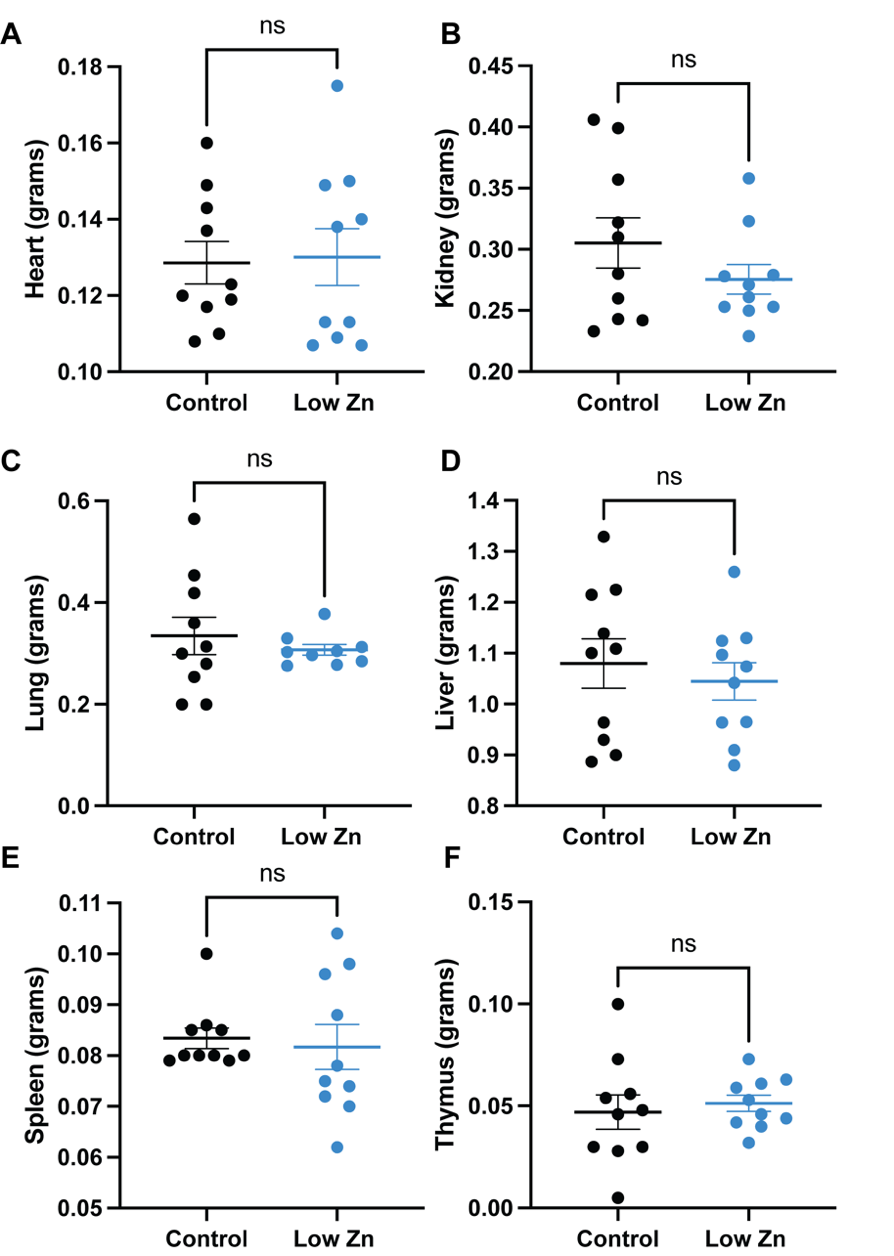
**

**Supplemental Figure 1. Organ weights from low Zn diet fed mice.** Organs were harvested and weighed following six weeks of control or low Zn diet. N = 10 / diet. All data are represented as mean ±SEM. Data in panels A-F analyzed by Student’s *t*-test. * *P* < 0.05, ** *P* < 0.01, *** *P* < 0.001, **** *P* < 0.0001.

**
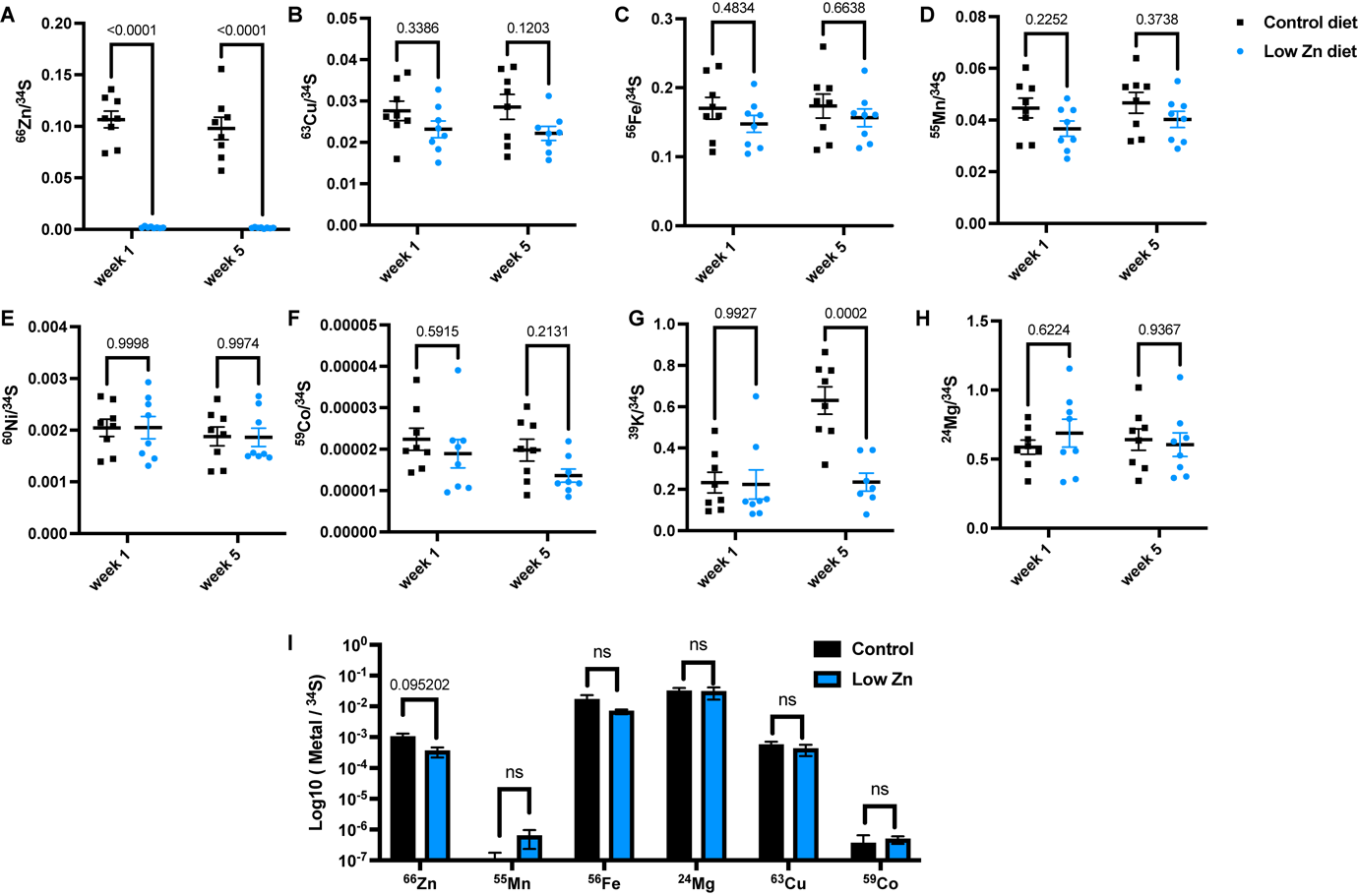
**

**Supplemental Figure 2. Fecal and serum metal levels of mice fed a control or low Zn diet.** Metal levels quantified from control or Zn deficient mice from feces (A-H) after one and five weeks of dietary intervention and serum (I) after six weeks of low Zn diet. Feces: N = 8 / diet, Serum: N = 4 / diet. All data are represented as mean ±SEM. Data in panels A-H analyzed by Two-way ANOVA with Tukey’s multiple comparisons test. Data in panel I analyzed by Student’s *t-*tests. * *P* < 0.05, ** *P* < 0.01, *** *P* < 0.001, **** *P* < 0.0001.


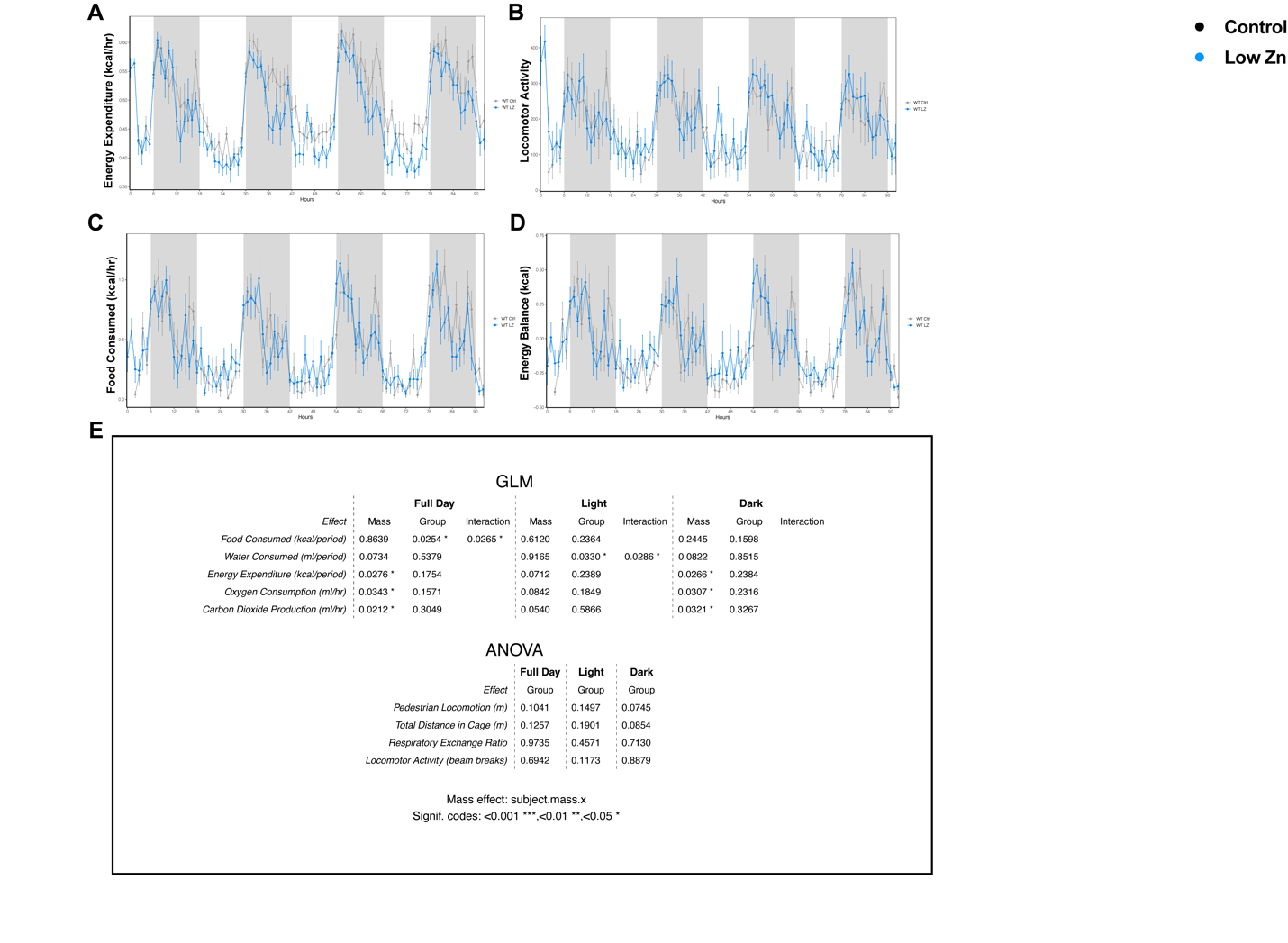


**Supplemental Figure 3.** **Metabolic cage analysis following one week of low Zn diet. (A)** Energy expenditure, **(B)** locomotor activity and **(C)** food consumption were measured to evaluate **(D)** energy balance in Zn-deficient mice following one week of diet (Panels A-D: light phase – white shading, dark phase – gray shading). N = 8 / diet. All data are represented as mean ±SEM.  **(E)** Weight adjusted statistical analysis of diet-dependent metabolic measures via CalR.

**
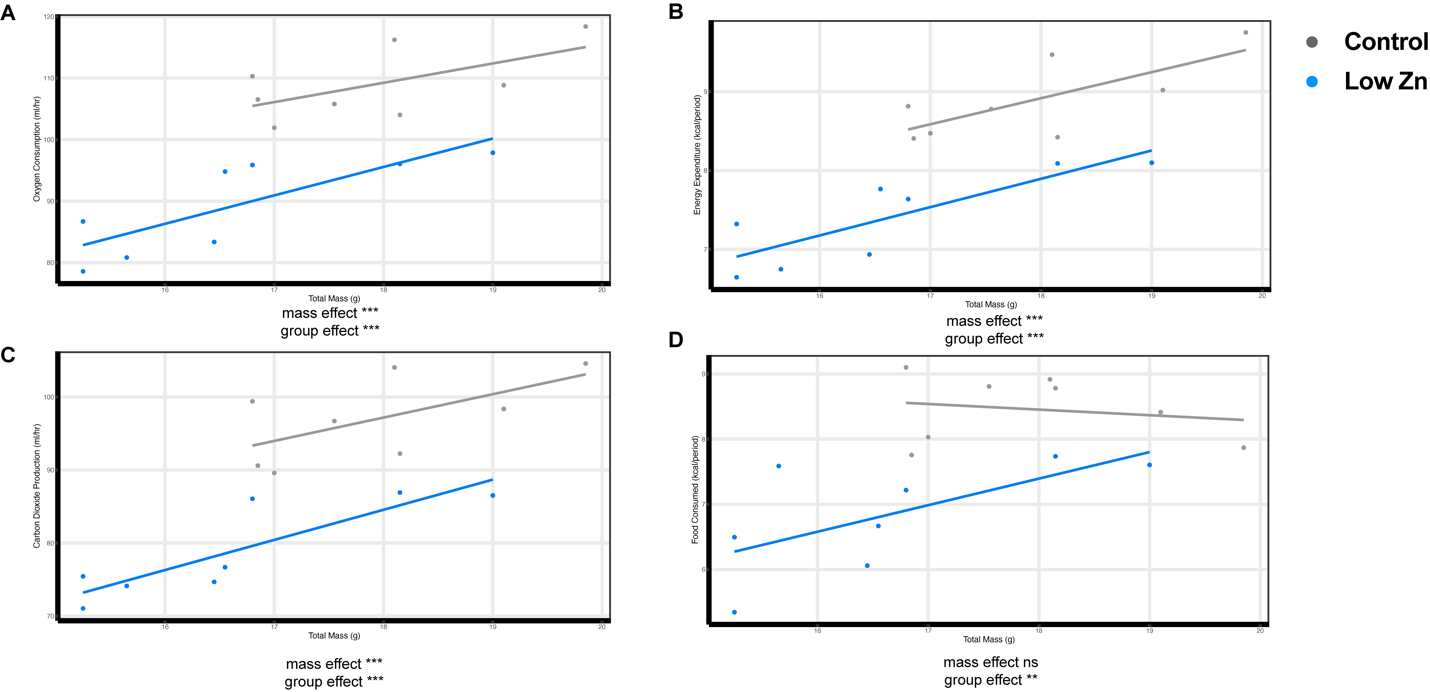
**

**Supplemental Figure 4.** **GLM based regression plots.** CalR analysis of group by mass interactions for (A) oxygen consumption, (B) energy expenditure, (C) carbon dioxide production, and (D) food consumption for mice in metabolic cages from weeks four to five of diet. N = 8 / diet. Slopes of control or low Zn-fed groups were used to determine appropriate statistical model. For variables that exhibit group-dependent mass interactions as demonstrated by slopes that are dissimilar between groups, ANOVA with interaction was performed. Otherwise, data were analyzed using GLM/ANCOVA.


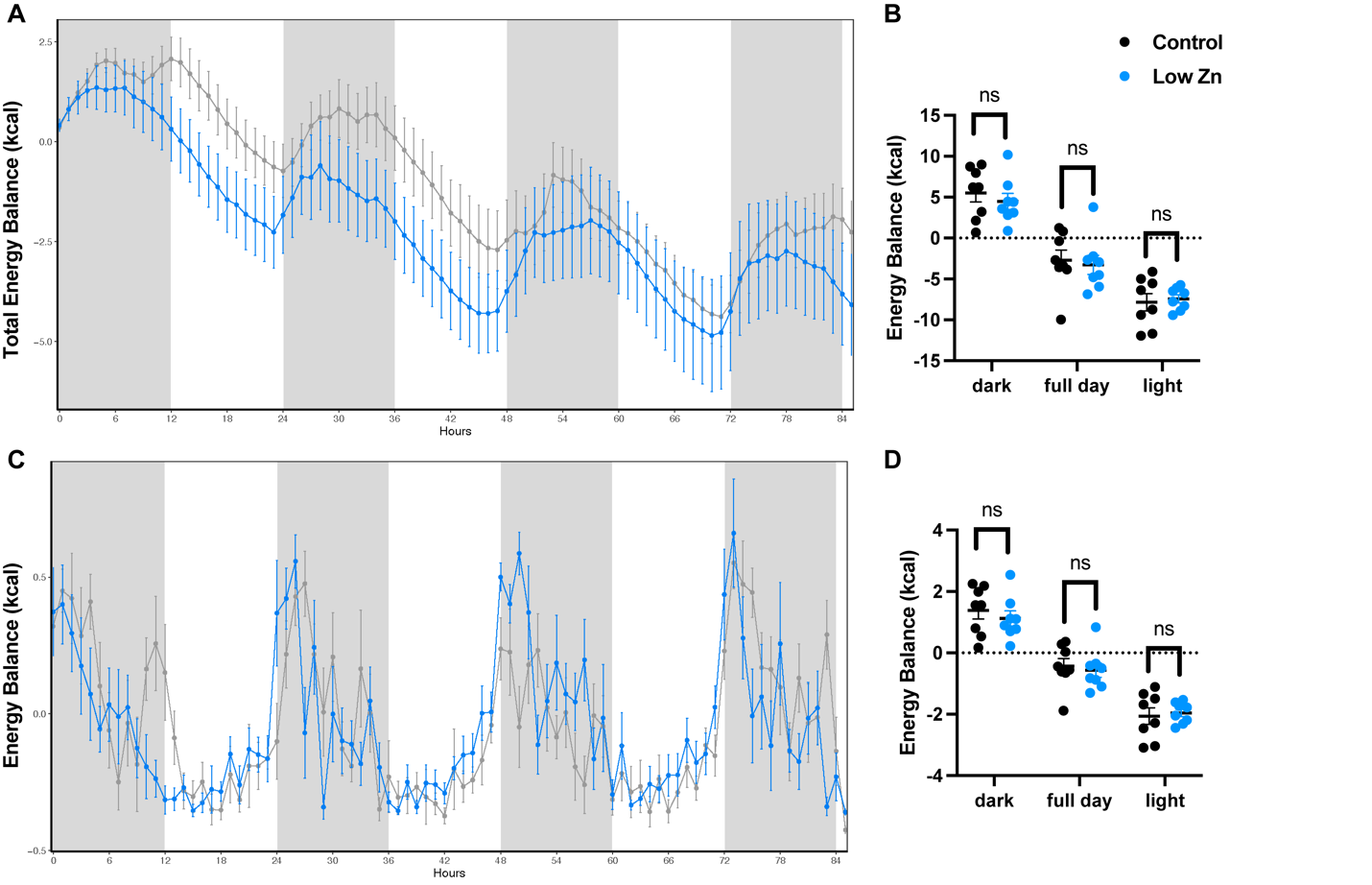


**Supplemental Figure 5. Energy balance of Zn-deficient mice**. **(A-D)** Energy balance was quantified from measurements of energy expenditure and energy intake (Panels A and C: light phase – white shading, dark phase – gray shading). N = 8 / diet. All data are represented as mean ±SEM. Statistical significance in panels B and D were determined by ANOVA model in CalR.
